# Smart Wearable Technologies for Balance Rehabilitation in Older Adults at Risk of Falls: Scoping Review and Comparative Analysis

**DOI:** 10.2196/69589

**Published:** 2025-05-28

**Authors:** Brooke Nairn, Vassilios Tsakanikas, Becky Gordon, Efterpi Karapintzou, Diego Kaski, Dimitrios I Fotiadis, Doris-Eva Bamiou

**Affiliations:** 1 Faculty of Brain Sciences, The Ear Institute, University College London, 332 Gray’s Inn Road, London, WC1X 8EE, United Kingdom, 44 07538640838; 2 Department of Neuro-otology, Royal National Ear Nose and Throat and Eastman Dental Hospital, University College London Hospitals, London, United Kingdom; 3 Department of Materials Science and Engineering, Unit of Medical Technology and Intelligent Information Systems, University of Ioannina, Ioannina, Greece; 4 Biomedical Engineering, University College London, London, United Kingdom; 5 Department of Clinical and Movement Neurosciences, Faculty of Brain Sciences, University College London, London, United Kingdom; 6 National Hospital for Neurology and Neurosurgery, University College London Hospitals, London, United Kingdom

**Keywords:** falls, balance, telerehabilitation, technology, augmented reality, motion tracking, stroke, mild cognitive impairment, long COVID, vestibular

## Abstract

**Background:**

Falls among older adults are a significant public health concern, often leading to severe injuries, decreased quality of life, and substantial health care costs. Smart wearable technologies for balance rehabilitation present a promising avenue for addressing the falls epidemic, capable of providing detailed objective movement data, engaging visuals, and real-time feedback. With the recent and rapid evolution of innovative technologies, including artificial intelligence (AI), augmented reality (AR) or virtual reality (VR), and motion tracking, there is a need to evaluate the market to identify the most effective and accessible smart balance systems currently available.

**Objective:**

This study aims to evaluate the current landscape of smart wearable technology systems for balance rehabilitation in older adults at risk of falls. In addition, it aims to compare market-available systems to the telerehabilitation of balance clinical and economic decision support system (TeleRehab DSS), a recently developed smart balance system.

**Methods:**

A scoping review and strengths, weaknesses, opportunities, and threats (SWOT) analysis was completed, exploring the landscape of smart balance systems in older adults at risk of falls. Following the Preferred Reporting Items for Systematic Reviews and Meta-Analyses extension for Scoping Reviews (PRISMA-ScR) guidelines, electronic databases PubMed, MEDLINE, and Cochrane were systematically searched for articles in English from July 1, 2014, to July 1, 2024. Gray literature searches of relevant institutions and web pages were also conducted. The database search and commercial systems were then compared against the TeleRehab DSS in a SWOT analysis.

**Results:**

The scoping review yielded 17 systems that met the inclusion criteria; 10 investigational systems and 7 commercially available systems. Out of 10 studies, only 1 reported the use of intelligent learning or AI, 8 studies reported the use of motion tracking, and 9 studies used virtual reality. Of the studies incorporating motion tracking, 3 provided feedback as either visual or auditory. All but 2 studies reported the use of gamification, and 7 studies incorporated balance exercises. In total, 2 studies reported remote delivery, with 5 being clinician-supervised and 4 providing a clinician report. The SWOT analysis of TeleRehab DSS against the 7 market-available smart balance systems revealed several unique advantages, including personalized therapy with AI-DSS, AR for real-world interaction, enhanced clinician involvement, and comprehensive data analytics.

**Conclusions:**

The findings from this scoping review highlight the rapid evolution of smart balance systems, yet significant gaps remain in AI integration, remote accessibility, and clinician-driven data analytics. Despite limitations such as cost, accessibility, and user training requirements, TeleRehab DSS emerges as a significant innovation, addressing many of these gaps through AI-driven personalization, AR for real-world interaction, and real-time clinician monitoring. These features position it as a next-generation solution that aligns closely with the evolving needs of patients and clinicians. The results of this review provide valuable insights for future research, supporting the need for further validation studies and the development of more intelligent and accessible balance rehabilitation technologies.

## Introduction

### Background

Falls among older adults are a significant public health concern, often leading to severe injuries, decreased quality of life, and substantial health care costs [1-4]. Globally, falls are the second leading cause of accidental or unintentional injury deaths, with adults over 65 years being the most affected group [1-4]. Each year, approximately 28%‐35% of people aged 65 years and over experience a fall, increasing to 32%‐42% for those over 70 years [1-3]. This rising trend underscores the urgent need to address balance disorders and implement effective fall prevention strategies.

The impact of falls and balance disorders extends beyond individual health, affecting families, communities, and health care systems. Individuals who experience falls often face prolonged recovery periods, reduced independence, and heightened fear of falling again, which can lead to social isolation and decreased physical activity [1,2,5,6]. Communities bear the emotional and financial burdens of caring for fall-prone older adults, while health care systems are strained by the high costs associated with emergency treatments, hospitalizations, and long-term care needs. Consequently, there is a critical need for effective interventions that can mitigate these negative effects, with health care services that are accessible and meet the needs of older adults.

### Previous Work

Smart wearable technology for balance rehabilitation presents a promising avenue for addressing the falls epidemic among older adults [7-11]. These advanced systems, often prescribed by health care professionals have the potential to facilitate home-based balance exercises by leveraging motion tracking, virtual reality (VR) or augmented reality (AR), and real-time feedback [10-14], Emerging evidence suggests that these digital health technologies, when combined with personalized training and regular home-based practice, can improve balance and gait outcomes, thereby reducing the risk of falls among older adults [10-12,15]. The World Health Organization defined digital health as “the field of knowledge and practice associated with the development and use of digital technologies to improve health” [16-18]. These technologies include both assistive technologies designed to maintain or improve the independence, social participation, and functionality of older people at home, as well as health information technology for managing long-term conditions, including telehealth, wearable devices, and mobile health [16-18].

The adoption of wearable technology has increased significantly in recent years. In Germany, a 2016 survey found that 33% of adults use a wearable, with 57%‐63% reporting a willingness to use wearables with health monitoring sensors [19]. In the United States, it is estimated that 1 in 3 American adults uses a wearable device such as smart watch or band to track their health and fitness [20]. However, despite this growth, challenges remain in widespread adoption among older adults. A survey in the United Kingdom indicated that many seniors over 65 years are hesitant to adopt new technologies due to concerns about online privacy, high costs, and the rapid pace of technological advancements [21,22], with similar findings reported in Singapore [23]. These statistics highlight the need to evaluate the market and identify the most effective and accessible smart balance systems currently available for older adults.

These wearable technologies have the ability to enhance multisensory stimulation, a key component required for balance [24]. Multisensory integration, that is, the integration of visual, vestibular, and somatosensory inputs, is critical for bodily awareness and movement coordination [24]. Technologies such as AR- and VR-supported systems can enhance rehabilitation by delivering immersive, and multisensory experiences, offering a strong advantage over conventional balance interventions [24].

Despite the growing number of smart balance systems available, many have not been systematically compared in terms of suitability for older adults. Previous reviews have focused on general rehabilitation technologies [10,14,25,26], but no study has comprehensively evaluated these systems against predefined criteria such as AI integration, remote monitoring, and clinical usability. To address this gap we establish clear inclusion or exclusion criteria to assess the most clinically viable smart balance systems for older adults, enabling clinicians to make evidence-based decisions in the selection of rehabilitative technology.

### Research Question and Aims

This scoping review aims to address this gap, by summarizing the current landscape of smart wearable technologies for balance rehabilitation in older adults at risk of falls. Throughout this study, smart wearable technologies refer to systems that incorporate motion tracking and some form of visual display (ie, computer interface, VR, or AR). The review seeks to provide clinicians with a comprehensive understanding of available resources, facilitating informed decision-making of rehabilitative technologies for balance.

In addition, we compare these existing systems to the TeleRehab Decision Support System (TeleRehab DSS) [27], a novel tool designed to reduce fall risk in individuals with balance problems or dizziness through: (1) AI analytics for personalized rehabilitation, (2) remote clinician monitoring for real-time assessment, and (3) multisensory balance rehabilitation with real-time patient feedback.

**Figure 1.**
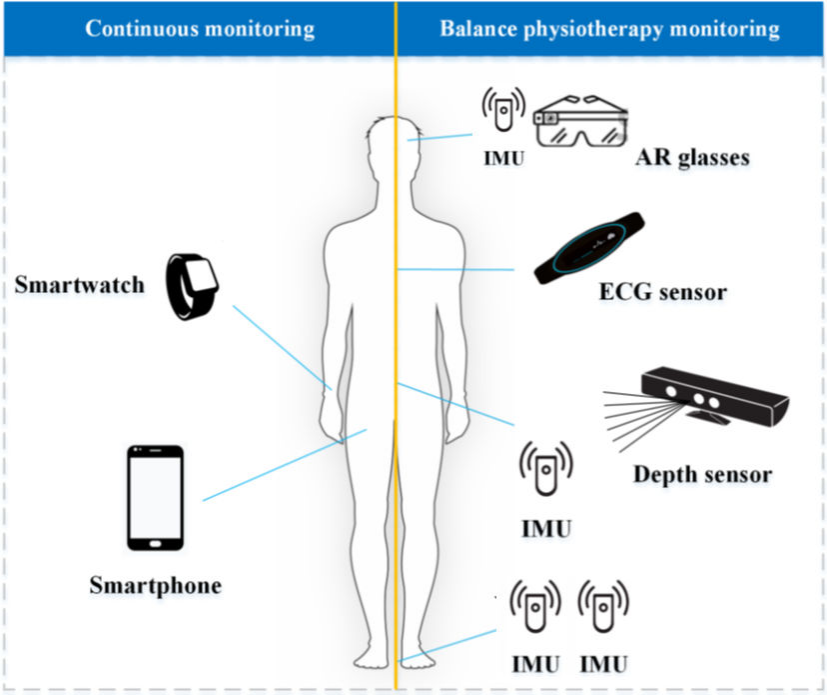
TeleRehab Decision Support System (TeleRehab DSS) equipment configuration including an augmented reality head-mounted display, smartwatch, smartphone, 4 IMU (head, trunk, and ankles), a chest ECG monitor, and depth camera. ECG: electrocardiogram; IMU: inertial measurement unit.

TeleRehab DSS is a next-generation AI-driven balance therapy platform that integrates depth-sensing body-worn motion trackers, heart-rate monitors, and AR interfaces to deliver highly personalized home-based rehabilitation (Figure 1). By conducting a strengths, weaknesses, opportunities, and threats (SWOT) analysis [28,29], this study compares TeleRehab DSS against other market solutions and investigation systems, identifying it is unique advantages and areas for improvement.

The primary research questions of this scoping review and SWOT analysis are: (1) to identify the current commercially available or investigational smart wearable technologies for balance rehabilitation that support clinicians in managing older adults with balance disorders or dizziness at risk of falls, and (2) to conduct a comparative analysis evaluating how these solutions compare to TeleRehab DSS.

This work aims to raise awareness of available technology-based rehabilitation solutions, guide clinicians in selecting appropriate interventions, and, thus, contribute to reducing fall risk and improving the quality of life for older adults.

## Methods

### Study Design

This study combines a scoping review and a SWOT analysis–based market analysis to explore the landscape of smart wearable technologies for balance rehabilitation in older adults at risk of falls. Scoping reviews have flexible study designs that allow authors to include any type of study that may be appropriate to answer their research questions [30,31]. The scoping review identifies existing solutions and research trends, while the SWOT analysis evaluates market opportunities and challenges.

### Scoping Review

The scoping review was conducted to identify the current and emerging market landscape and for smart technology, including motion tracking, systems, or solutions for balance rehabilitation in older adults at risk of falls. The review follows the methodological approach proposed by Arksey and O’Malley [31], which consists of six stages: (1) identifying research questions; (2) identifying relevant studies; (3) study selection; (4) charting the data; and (5) collating, summarizing, and reporting the results; and (6) consultation with stakeholders. The Preferred Reporting Items for Systematic Reviews and Meta-Analyses extension for Scoping Reviews (PRISMA-ScR) guidelines were followed (Checklist 1) [30].

### Search Strategy

Three main concepts were identified from the review question: (1) balance disorders, (2) remote rehabilitation, and (3) wearable technology solutions or systems. For each concept, subject headings were used, and when possible, keywords were searched using synonyms and related terms. A systematic search of PubMed, MEDLINE, and Cochrane databases for articles in the English language from July 1, 2014, to July 1, 2024, was conducted. This timeframe allowed the review to capture the early emergence of modern smart wearables until the more recent advancements, which are relevant to current practice, while excluding older, less relevant technologies. Articles were included if they described completed studies, ongoing studies, or protocols for interventions related to smart wearable technologies for balance rehabilitation in older adults at risk of falls. This criterion was selected in efforts to address this identified gap of smart wearable technology among older adults at risk of falls, specifically. Please refer to Multimedia Appendix 1 for details of the search strategy. The search strategies were developed through discussion (BN and BG) and with the aid of an experienced researcher (DEB).

A gray literature search was conducted using Google and targeted searches within company web pages, professional organizations, industry leaders, and clinical trial registries to identify relevant reports, guidelines, and nonpeer-reviewed sources. Search queries included terms related to smart wearable balance rehabilitation, AI, and remote monitoring, with results filtered based on targeted population group, publication date (July 1, 2014, to July 1, 2024), and relevance to remote balance rehabilitation. Only reports from reputable institutions, research organizations, and technology companies were included, while opinion pieces and promotional content were excluded.

Finally, consultation with a stakeholder (Health Innovation Network) was carried out to identify and discuss additional references and insights beyond those retrieved in the literature.

### Eligibility Criteria

Studies were included if the system was a technological system or solution with wearables and motion tracking for balance rehabilitation to improve balance or gait outcomes or reduce falls in older adults at risk of falls. The population of interest included older adults (≥50 y) with potential balance impairments or falls deficits due to age-related physiological decline or specific health conditions. While an explicit fall risk assessment (eg, Timed Up and Go Test and Berg Balance Scale) was not a requirement for study inclusion, the selected studies targeted populations commonly identified in the literature as at risk of falls. These included individuals with neurological conditions (eg, stroke, Parkinson disease, and mild cognitive impairment), vestibular dysfunction, orthopedic conditions affecting mobility, or generalized age-related balance impairments. Studies had to be published after July 1, 2014, and in the English language. Protocols were included to capture ongoing developments and emerging trends in the field, as they provide insights into planned interventions and methodologies. Refer to Table 1 for inclusion and exclusion criteria.

**Table 1.** Inclusion and exclusion criteria of smart wearable technology systems for balance rehabilitation among older adults at risk of falls.

	Included	Excluded
Populations of interest	Community dwelling adults (50‐80 y) at risk of falls or dizziness	<50 or >80 years of age Without balance problems or not at risk of falls
Primary outcome	Primary outcome to improve balance or reduce risk of falls	Primary outcomes that did not assess balance and falls risk
System specification	Technology solutions to facilitate home based balance exercise intervention with motion tracking	Solutions which do not include motion tracking Systems which only look at gait via exoskeletons or robot assisted walking Systems involving only electrical stimulation or biofeedback Only assessed falls detection without rehabilitation
Prescription	Interventions prescribed by a health care professional	Systems not prescribed by a health care professional
Study design	Primary study design, such as RCTs^a^ and nonrandomized controlled trials, case studies, protocols	Systematic reviews and meta-analysis
Publication dates	July 2014-July 2024	Studies >10 years old
Publication language	English	Not English

a RCT: randomized controlled trial.

### Article Screening and Data Extraction

Database searching was completed by 2 authors (BN and BG). EndNote (Clarivate) was used to manage the literature retrieved, and after removing the duplicates using EndNote, the eligibility of papers was independently reviewed by 2 authors (BG and BN) at each key step, including abstract screening and full-text review. Lists of article selection by each author will be compared for agreement, and any controversial papers were further assessed by the third author (DEB) for the final decision.

One reviewer (BG) independently extracted data into a predesigned data extraction table, developed to systematically assess smart balance rehabilitation systems based on key technological, clinical, and research-based parameters. The table included: company or institution, system summary, population of use, current use of system, automated feedback or real-time monitoring, AI analytics, clinician report, motion tracking method, display features, gamification, balance exercises, user experience, and remote suitability. These categories were selected to capture essential features relevant to balance rehabilitation and to enable direct comparison with the TeleRehab DSS system. Study characteristics were also extracted, including authors, publication date, study design, country or region, to ensure standardization across included studies. For protocols, additional data on study objectives, planned interventions, target population, outcome measures, and projected timelines were recorded separately to identify trends in ongoing research. The structured table facilitated both the descriptive summary and the SWOT analysis, ensuring a comprehensive evaluation of available and investigational systems. Any disagreement in the data extracted from studies was resolved through discussion between reviewers.

A comparative analysis was then performed, focusing on key aspects of technological features (inclusion of AI, AR or VR, motion tracking, and remote monitoring), rehabilitation focus (types of balance exercises, gamification, and feedback mechanisms), and clinical integration (mode of clinical supervision, reporting, and remote accessibility).

### Synthesis of Results

The reporting of key subsections and information throughout this manuscript follows the PRISMA-ScR guidelines. Given that no dedicated reporting guidelines exist for SWOT analyses within the Enhancing the Quality and Transparency of Health Research network, an adapted approach was taken to align with systematic review standards.

To provide a structured comparison, the Results section was organized into two primary classifications: (1) smart balance systems currently under investigation, further divided by study type (randomized controlled trials, quasi-experimental studies, feasibility and usability studies, and study protocols), and (2) commercially available smart balance systems, with an evaluation of their features, accessibility, and evidence base.

### Comparative Analysis

A structured comparative analysis was conducted to evaluate the features and functionalities of smart balance rehabilitation systems in relation to the TeleRehab DSS. Systems were assessed based on key technological (motion tracking, AI integration, real-time feedback, display modality, and gamification), clinical (target population, rehabilitation exercises, clinician oversight, and report generation), and usability parameters (remote suitability and adaptability for home use, and patient engagement features).

Data for these comparisons were extracted systematically from published literature, company websites, and clinical trial protocols and summarized in a descriptive manner. Where full-text articles or system specifications did not explicitly mention certain features (eg, AI support and gamification), they were marked as “not reported” rather than assumed to be absent. The competitive features of each study are listed in Table 2 and Table 3 to enable a comparative assessment of the TeleRehab DSS with investigational and commercially available systems. The study characteristics of the investigational and commercially available systems are collated in the Multimedia Appendix 2.

**Table 2.** Competitive features of the included studies.

Author (year)	AI^a^ supported	Motion tracking	Real-time feedback	AR^b^ or VR^c^	Gamification	Balance exercises	Remotely delivered	Clinician supervised	Clinician report
Liao et al [32]		✓	✓	✓	✓	✓		✓	✓
Proffitt et al [33]		✓		✓	✓		✓		
Rosiak et al [34]		✓	✓	✓	✓	✓			
Khushnood [35]		✓		✓	✓	✓		✓	
Ku et al [36]		✓		✓	✓	✓		✓	
Bessa [37]				✓		✓		✓	
da Silva Soares [38]				✓	✓	✓			
Yun et al [39]	✓	✓		✓					✓
Zeigelboim et al [40]		✓		✓	✓	✓		✓	✓
Guo et al [15]		✓	✓		✓		✓		✓

a AI: artificial intelligence.

b AR: augmented reality.

c VR: virtual reality.

**Table 3.** Competitive features of the included commercial systems.

Company	AI^a^ supported	Motion tracking	Real-time feedback	AR^b^ or VR^c^	Gamification	Balance exercises	Remotely delivered	Clinician supervised	Clinician report
TeleRehab DSS^d^	✓	✓	✓	✓	✓	✓	✓	✓	✓
HoloBalance	✓	✓	✓	✓	✓	✓	✓	✓	✓
Reflexion Health VERA		✓	✓		✓	✓	✓	✓	✓
XRhealth		✓	✓	✓	✓	✓	✓	✓	✓
Evolv Rehab	✓	✓	✓		✓	✓	✓	✓	✓
Jintronix		✓	✓		✓	✓	✓	✓	✓
TRAK	✓	✓	✓			✓	✓	✓	✓
Homebalance		✓	✓		✓	✓	✓	✓	✓

a AI: artificial intelligence.

b AR: augmented reality.

c VR: virtual reality.

d TeleRehab DSS: TeleRehab Decision Support System.

### SWOT Analysis

The SWOT analysis was performed to contextualize the competitive positioning of TeleRehab DSS in relation to the evaluated systems. Originally developed as a business strategy tool, SWOT analysis is widely used to compare an organization, product, or service against competitors by identifying internal (strengths and weaknesses) and external factors (opportunities and threats) [29]. Beyond business applications, SWOT analysis has been extensively applied in health research, including health care policy and health technology assessment [28,41,42].

For instance, in the United States, a SWOT analysis of the health care industry IT adoption identified key challenges in improving patient safety, data security, cost containment, and productivity [28]. This demonstrates the value of SWOT analysis in evaluating digital health innovations and their impact on health care systems.

In this study, a SWOT analysis was conducted to evaluate the positioning of TeleRehab DSS relative to investigational and commercial smart balance systems. The SWOT structured approach was applied (Textbox 1).

This SWOT analysis was structured based on established methodologies from previous digital health and medical technology SWOT frameworks [28,29,41,42]. The results are summarized in Textbox 2 and further contextualized in the discussion to provide key insights for clinicians, researchers, and stakeholders.

Textbox 1. **SWOT (strengths, weaknesses, opportunities, and threats) summary criteria of TeleRehab Decision Support System (TeleRehab DSS).**

- Strengths: unique features of TeleRehab DSS not present in the reviewed systems, such as AI-driven personalization, real-time monitoring, and augmented reality based balance exercises.
- Weaknesses: challenges impacting usability, accessibility, or scalability, including cost, technical proficiency requirements, and need for external validation.
- Opportunities: emerging trends in digital rehabilitation, such as the growing adoption of remote balance training and increasing demand for AI-driven clinical support.
- Threats: competing technologies with overlapping features, regulatory hurdles, and user adoption challenges.

Textbox 2. **SWOT (strengths, weaknesses, opportunities, and threats) analysis of TeleRehab Decision Support System (TeleRehab DSS): positioning in the smart balance technology landscape.**

**Strengths**

- Integrated multisensory feedback: combines motion tracking, heart rate, and AR for a detailed and engaging rehabilitation experience, offering richer patient interaction than many single-feature systems.
- Evidence-based: as TeleRehab DSS is the next iteration of HOLOBalance, it is an evidence-based system with initial preliminary findings supporting its feasibility and acceptability in older adults at risk of falls.
- AI–powered personalization: customizes exercises and therapy progression based on individual patient data, making it more adaptive and individualized than traditional preset programs.
- Real-time clinician monitoring: allows clinicians to monitor patient performance remotely and in real-time, enhancing oversight and intervention capabilities.
- Comprehensive data analytics: enables in-depth tracking of patient progress, which can aid in refining treatment plans and provide valuable data for ongoing research.
- Enhanced accessibility: offers home-based therapy, reducing the need for in-person sessions, which is especially beneficial for patients with limited mobility or those in remote areas.

**Weaknesses**

- High initial cost: advanced sensors and AR interfaces increase upfront expenses, potentially limiting accessibility in low-resource settings.
- Complex setup and maintenance: multicomponent setup can be challenging for non-tech-savvy users or those with limited technical support.
- Potential for technological resistance: some older adults or less tech-savvy patients may struggle with advanced AR or sensor-based systems, leading to reluctance or inconsistent usage.
- Dependence on internet connectivity: real-time features require stable internet, limiting use in areas with poor or unreliable connections.

**Opportunities**

- Expansion into related rehabilitation areas: the system’s adaptability makes it suitable for other types of rehabilitation (eg, poststroke or musculoskeletal recovery), expanding its market potential.
- Growing demand for telemedicine solutions: the increase in telemedicine adoption, driven by the need for remote health care solutions, offers a supportive environment for TeleRehab DSS’s expansion.
- Collaboration with wearable technology companies: partnering with popular wearables (like Fitbit [Google] or Apple Watch) could expand monitoring capabilities and potentially reduce system costs.
- Inclusion in health care insurance plans: efforts to integrate such technology into insurance plans could drive affordability and adoption by reducing the cost burden on patients.

**Threats**

- Intense market competition: competing solutions like XRHealth, Jintronix, and Reflexion Health, each with specific strengths, may limit market share, especially if they are more affordable or easier to use.
- Regulatory and compliance challenges: adhering to health care regulations (eg, Health Insurance Portability and Accountability Act and General Data Protection Regulation) is complex and costly, potentially slowing down deployment in new regions.
- Privacy and security concerns: handling sensitive health data brings stringent privacy requirements, and any breach could impact the system’s reputation and user trust.
- Adoption hurdles among clinicians and patients: clinicians may be skeptical of the efficacy of remote rehabilitation, and some patients may prefer traditional, in-person therapy over telemedicine solutions.

## Results

### Overview

**Figure 2.**
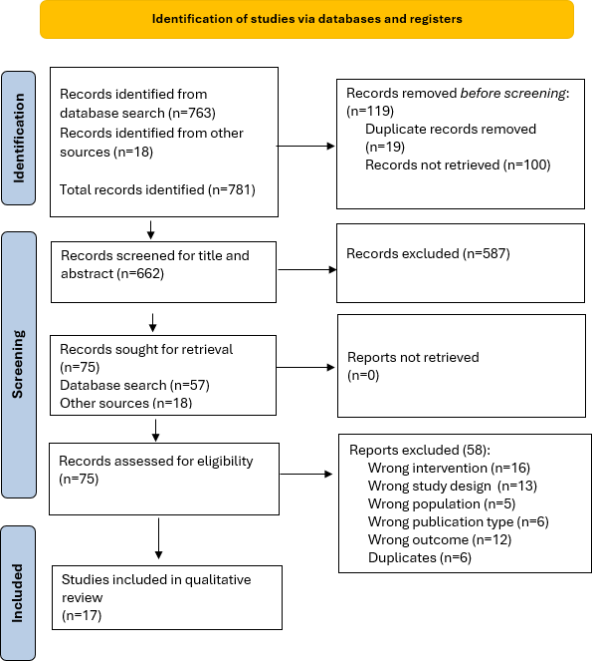
Preferred Reporting Items for Systematic Reviews and Meta-Analyses (PRISMA) flow chart of identification and selection of studies.

The systematic search yielded 626 articles after removing duplicates (Figure 2). Of these, 57 were screened in full text, and subsequently, 10 studies met the inclusion criteria. In addition, manual retrieval of 18 market available systems was identified by searching the gray literature and institution web pages, with 7 included.

### Included Study Characteristics

#### Overview

The characteristics of the included papers identified by database search that are described in the supplementary material. The 10 studies identified from the database search were published between 2015‐2023, with 3 from Brazil, 2 from Korea, 1 from Pakistan, 1 from the United States, 1 from China, 1 from Taiwan, and 1 from Poland.

Data analysis revealed various smart balance rehabilitation systems currently being researched, that is, those using motion tracking and some form of visual display, with details found in the supplementary materials (Multimedia Appendix 1).

In total, 5 [15,33,35,37,38] of the included studies investigated the use of smart balance systems in stroke patients; 1 study [34] with peripheral vestibular dysfunction patients, 1 study [32] looked at patients with Parkinson disease, 1 [36] in independently mobile older adults, 1 [40] in adults with hereditary spastic paraplegia and 1 [39] with physiatrists, occupational therapists and older adults with mild cognitive impairment.

#### Intervention Features

Multimedia Appendix 2 details the intervention features of the included studies. Only 1 study [39] reported the use of intelligent learning or AI. In total, 8 studies [15,32-36,39,40] reported use of motion tracking, with 2 protocols [37,38] not reporting the use of motion tracking. Of the studies incorporating motion tracking, 3 [15,32,34] made comments of feedback provided as either visual or auditory. A total of 9 [32-40] of the 10 included studies incorporated either AR or VR, with 1 study [15] using a computer interface. All [15,32-36,38,40] but 2 studies [37,39] reported use of gamification (the addition of game elements to nongame activities; ie, Apple picking game added to the activity of bending over), and 7 [32,34-38,40] studies incorporated balance exercises. In total, 2 studies [15,33] reported that it was remotely delivered, with 5 studies [32,35-37,40] being clinician supervised and 4 [15,32,39,40] providing a clinician report.

### Comparative Analysis of TeleRehab DSS and Smart Balance Systems Under Investigation

#### Overview

The rapid advancement of AI, AR or VR, motion tracking, and remote monitoring has led to the development of multiple smart balance rehabilitation systems, necessitating an analysis of these systems from both a competitive viewpoint as well as to identify key factors for consideration in future work. This review identified 10 investigational systems, including 5 registered protocols, 3 randomized controlled trials, 1 quasi-experimental study, and 1 feasibility or usability study. The target populations of these studies ranged from stroke survivors [15,33], older adults with balance impairments [36], patients with Parkinson disease [32], individuals with mild cognitive impairment [39], to those with vestibular dysfunction [34].

Most of the investigational systems share common features, including motion tracking and gamification, with a strong emphasis on VR-based rehabilitation. However, none integrates all the key technological components present in TeleRehab DSS, particularly AI-driven personalization, AR-based balance training, real-time corrective feedback, and comprehensive clinician monitoring.

#### Motion Tracking and Gamification

While most investigational systems use motion sensors, their level of data granularity and clinical application varies. All but 2 studies [35,38] used motion tracking via inertial measurement units (IMUs), depth cameras, or gaming sensors (eg, Xbox Kinect and Nintendo Wii). Guo et al [15] used 3 IMUs for feedback on static and dynamic balance in stroke patients but lacked real-time corrective feedback. TeleRehab DSS differentiates and strengthens itself by using 4 IMUs and a depth camera, enabling a more detailed biomechanical analysis than existing systems.

Gamification was incorporated in 8 of the 10 studies, reinforcing its growing role in smart rehabilitation technologies. However, the extent of engagement and customization varied with limited details of the games provided. The consistent presence of gamification highlights its importance, which TeleRehab DSS acknowledges for enhancing engagement through customization and interactive exercises.

#### AR versus VR

VR was used by all studies (n=9) [32,33,35-40,43] delivered via a head-mounted display (HMD) or screen-based interfaces, with one study using AR [36]. Liao et al [32] explored VR rehabilitation for Parkinson disease, but primarily for upper limb motor function rather than balance and gait rehabilitation. Zeigelboim et al [40] combined VR and vestibular rehabilitation using Nintendo Wii Balance Board and hand-held IMUs, providing a promising but non–AI-enhanced system.

TeleRehab DSS is distinct in its use of AR rather than VR, facilitating real-world applicability for balance training and improved safety through an overlay of one’s environment, unlike VR-based systems that require simulated environments that encompass the entire visual field.

#### AI and Real-Time Feedback

Few systems incorporate AI for adaptive rehabilitation. The one study that did report AI personalization [39] focused more on cognitive training rather than balance rehabilitation. Furthermore, only 3 [15,32,34] studies reported providing feedback, which was either visual or auditory, with no mention of real-time corrective feedback provided, such as the TeleRehab DSS.

The AI personalization stands out as a unique and key advantageous feature of TeleRehab DSS [39]. Providing tailored rehabilitation plans has always been recognized as a gold standard, and TeleRehab DSS has the potential to enhance this personalization with real-time corrective feedback and dynamic adjustments, predictive analytics, and objective data to optimize outcomes.

#### Remote Usability and Clinician Involvement

Many investigational systems are clinician-supervised in clinical settings, with limited capability for remote rehabilitation. Only 2 studies [33,35] explicitly support home-based rehabilitation, while others provided delayed reporting to clinicians rather than real-time oversight. TeleRehab DSS is fully designed for remote use, with real-time clinician monitoring, remote dashboards, and data-driven decision-making, reducing travel time and [33,39] costs for both patients and clinicians.

### Comparative Analysis of TeleRehab DSS and Commercial Smart Balance Systems

#### Overview

The increasing prevalence of fall-related injuries among older adults has driven the rapid advancement of smart balance rehabilitation systems. TeleRehab DSS offers a multifaceted approach to telerehabilitation, integrating AI-driven adaptive therapy, AR-based exercises, gamification, and real-time clinician monitoring to enhance patient engagement and rehabilitation outcomes. This section compares TeleRehab DSS with commercially available systems, focusing on technology integration, monitoring capabilities, and user engagement strategies.

#### AR-Based Rehabilitation Systems

HOLOBalance [44] integrates AR and wearable sensors to deliver balance rehabilitation exercises, supported by a decision-support system for clinicians. It offers personalized interventions based on real-time sensor data, focusing primarily on fall prevention in older adults. While HOLOBalance provides a robust, AR-driven approach to balance training, TeleRehab DSS extends these capabilities with enhanced AI-driven personalization and broader functionality with gamification, multisensory feedback, and a more comprehensive decision-support system beyond fall prevention. Furthermore, as TeleRehab DSS is the next iteration of HOLOBalance, it is an evidence-based system with initial preliminary findings supporting its feasibility and acceptability in older adults at risk of falls [45].

Reflexion Health’s VERA [46] uses motion capture technology to guide patients through balance and rehabilitation exercises, offering real-time feedback to improve exercise accuracy and efficacy. Its remote monitoring capabilities allow clinicians to oversee patient progress without requiring frequent in-person visits, however, it lacks AR and AI-driven personalization, limiting adaptability for diverse rehabilitation needs. TeleRehab DSS leverages AR and AI-driven personalization for enhancing patient engagement, treatment adherence, and the overall rehabilitation experience.

#### VR-Based Rehabilitation Systems

XRHealth [47] and Evolv Rehab [48] use VR environments for immersive therapy and gamification with data analytics and clinician oversight. While VR is effective, TeleRehab DSS’s AR integration offers the advantage of real-world applicability, improving the transferability of balance skills to daily life. Furthermore, TeleRehab DSS’s AI-driven personalization provides more tailored interventions compared to XRHealth’s reliance on preset VR experiences. [48]

#### Gamified and Motion-Tracked Rehabilitation Systems

Jintronix [49] integrates motion capture and gamification to create an interactive rehabilitation experience, using gamified exercises to boost patient adherence and enjoyment, particularly for long-term therapy. Its remote monitoring capabilities allow clinicians to oversee progress and adjust therapy as needed, offering flexibility for remote rehabilitation. However, Jintronix’s approach relies on preset gamified content and lacks the AI-driven personalization of TeleRehab DSS. TeleRehab’s emphasis on real-time data analytics and adaptive clinician monitoring enables more precise and tailored interventions, making it a more comprehensive solution for rehabilitation.

TRAK [50] is a telerehabilitation platform that provides tailored exercise plans with video guidance, offering a user-friendly interface suited for patients with minimal technical skills. It demonstrates exercises through videos to ensure proper form and tracks patient progress, generating reports for clinicians. However, it lacks real-time feedback and monitoring. In comparison, TeleRehab DSS offers a more comprehensive solution with multisensory feedback, real-time clinician oversight, and adaptive support, making it particularly effective for complex rehabilitation cases requiring continuous adjustments.

Home Balance [51] provides basic balance exercises supported by instructional videos or written guidelines, offering a cost-effective solution accessible to a wide range of users. However, it lacks the technological sophistication of TeleRehab DSS, which includes features like motion tracking, real-time feedback, and AI-driven customization. While Home Balance is affordable, it may not meet the needs of users requiring intensive or guided therapy, where TeleRehab DSS’s advanced capabilities are more effective.

### Strengths of TeleRehab DSS

Following a comparative evaluation of the above smart balance systems for balance rehabilitation, several unique advantages of TeleRehab DSS become apparent: (1) personalized therapy with AI-DSS, (2) AR for real-world interaction, (3) enhanced clinician involvement, and (4) comprehensive data analytics.

TeleRehab DSS’s AI-based approach allows for highly personalized therapy, adapting to individual patient needs in real-time. This level of customization is not present in most other systems, which often rely on generic exercise plans and preset “level” progressions. Unlike VR-based systems that isolate patients in virtual environments, TeleRehab DSS’s AR functionality enables patients to interact with their real surroundings, enhancing the practical applicability of their training, while also improving safety. TeleRehab DSS offers clinicians a comprehensive view of patient progress through real-time monitoring and detailed data analytics uploaded to a remote dashboard, promoting a collaborative and adaptable rehabilitation process with big data objective analytics. The advanced analytics in TeleRehab DSS enable continuous monitoring of patient progress, allowing clinicians to make data-driven adjustments to therapy plans and flag concerns requiring prompt action. This feature distinguishes it from systems that provide only summary data or lack in-depth analytics.

Moreover, telerehabilitation enhances accessibility, allowing patients to engage in rehabilitation from home, reducing travel burdens, which can be a significant barrier for patients [52,53]. However, older adults with limited digital literacy or cognitive impairments may require additional support to engage effectively with technology-driven interventions. TeleRehab DSS addresses this by incorporating reminders, remote clinician supervision, structured guidance, and progressive task difficulty adjustments to accommodate varying user abilities. TeleRehab DSS can also facilitate regular virtual supervision with real-time feedback and monitoring by healthcare professionals, promoting improved adherence [52,53].

### Limitations of TeleRehab DSS

Although TeleRehab DSS presents itself as superior to the included studies, it also presents limitations. Its high-tech integration may be challenging for patients or clinicians with limited technical skills, and advanced technology of this nature may require a higher upfront investment compared to simpler systems. While promising, long-term clinical validation and comparative effectiveness studies are needed to establish its superiority conclusively.

Furthermore, in comparison to the commercially available systems, TeleRehab DSS may incur higher costs, complexity of use, set-up demands, and internet dependence.

### Summary

In comparison with other smart balance systems, TeleRehab DSS stands out as a highly adaptive and advanced smart balance system with remote balanced rehabilitation. It combines AI-driven personalization, AR-based real-world interaction, and comprehensive monitoring, creating a robust tool for balance rehabilitation in older adults at risk of falls. While cost and complexity may pose challenges, the potential benefits for patient outcomes, clinician oversight, and data-driven therapy adjustments underscore TeleRehab DSS’s value in the current market. By addressing these limitations, TeleRehab DSS can strengthen its position as a leading solution in balance rehabilitation and support a broader range of patients.

## Discussion

This scoping review and SWOT analysis identified 17 smart wearable technologies for balance rehabilitation in older adults at risk of falls, comprising 10 investigational systems and 7 commercially available solutions.

### Comparison With Investigational and Commercial Systems

Across the investigational systems, the findings highlight key trends among smart wearable technologies for balance rehabilitation, including the widespread integration of VR (9/10 studies), the reliance on motion tracking (8/10 studies), and gamification (8/10 studies), but limited adoption of AI-driven personalization (1/10 studies). In addition, clinician involvement varied, with only 5 studies reporting clinician supervision, and even fewer (4/10 studies) providing clinician reports. Remote accessibility remained limited, with only 2 studies incorporating fully remote delivery models.

Compared to commercial systems like XRHealth, Evolv Rehab, Reflexion Health’s VERA, Jintronix, and TRAK, TeleRehab DSS offers greater adaptability, clinician oversight, and AI-driven decision making. Unlike VR-based platforms such as XRHealth, its AR-based approach enhances real-world applicability. HOLOBalance shares some features, but TeleRehab DSS extends these with advanced AI personalization for broader populations

The comparative analysis revealed that, while many investigational and commercial systems integrate immersive environments and gamification, they often lack AI-based decision support, real-time feedback, and comprehensive remote monitoring—features that define the TeleRehab DSS. The SWOT analysis positioned TeleRehab DSS as an innovative system that enhances balance rehabilitation through AI-driven personalization, AR-based real-world interaction, and clinician-integrated monitoring.

However, usability challenges remain, particularly for older adults with lower digital literacy or mild cognitive impairments. While TeleRehab DSS provides clinician-guided monitoring, further adaptations may be necessary to ensure ease of use, including simplified user interfaces, voice-guided instructions, and adaptive difficulty levels. Cost, accessibility, and user training requirements also remain barriers to implementation, emphasizing the need for inclusive design strategies. Future research should focus on validating the long-term clinical effectiveness of AI-driven balance rehabilitation solutions and expanding accessibility for diverse populations.

### Comparison With Conventional Rehabilitation Interventions

While this review contrasts TeleRehab DSS with existing smart balance rehabilitation systems, it is also important to consider how it compares to traditional balance rehabilitation interventions. Conventional rehabilitation typically involves face-to-face sessions with a physiotherapist, in either an individual or group format, where balance exercises and modifications are manually guided in real-time [54-57]. This approach offers personalized and immediate feedback, which can be beneficial, however, participants are often left to complete exercises alone and unsupervised between therapy sessions with inadequate exercise progressions that do not address real-world situations such as dual-task training simulating everyday challenges [56,58-61]. Access barriers, including geographical constraints, high costs, and scheduling difficulties, may also contribute to reduced adherence among conventional rehabilitation interventions, particularly for older adults with mobility limitations [56,58-60]. Additionally, clinic-based therapy often relies on subjective assessments rather than continuous movement tracking, which may impact precision in monitoring progress over time. In contrast, TeleRehab DSS enhances accessibility by providing a home-based, AI-supported rehabilitation platform that allows for continuous motion tracking, real-time feedback, and remote clinician monitoring. The integration of AR-based exercises further supports real-world functional training, which may improve carryover to daily activities.

### Alignment With Previous Work

Our findings align with previous reviews [13,14,34,43], which highlight the role of VR, wearable motion sensors, and telerehabilitation for improving balance rehabilitation. TeleRehab DSS advances this field by integrating AI-driven personalization and AR-based exercises, addressing previous limitations of static exercise programs and limited clinician oversight [25,26]. Unlike traditional VR-based systems, such as XRHealth (XRHealth R&D Ltd) and Evolv Rehab (Evolv Rehabilitation Technologies SL), TeleRehab DSS emphasizes real-world interactions through AR, enhancing the transferability of rehabilitative exercises to daily activities. This aligns with the findings by Man et al [26], emphasizing the role of telerehabilitation in increasing therapy accessibility and adherence, particularly among older adults.

Additionally, the inclusion of protocols in this scoping review highlights ongoing advancements and emerging research priorities in the field of wearable balance rehabilitation. These protocols highlight future directions and may evolve to address existing gaps with more of the key features, posing as a potential competitive threat.

### Limitations

This study has some limitations that should be considered. First, the scope of the review was confined to English-language studies, potentially excluding relevant research in other languages. Second, if system features were not documented or could not be retrieved in the literature, they were considered as not included, potentially introducing bias. Third, the reliance on publicly available data and published literature for the analysis constrained the depth of the evaluation. Some systems may have additional proprietary features or unpublished validation studies that were not captured in this review. Fourth, the inclusion criteria focused on technologies with motion tracking and rehabilitation applications, excluding systems primarily aimed at fall detection or prevention without a rehabilitation component. While this was necessary to maintain the scope of the review, it may have excluded hybrid solutions with potential relevance. Finally, TeleRehab DSS is still in its early stages of implementation and validation, and therefore its evaluation is based on theoretical potential rather than long-term empirical data.

These limitations highlight the need for multilingual reviews, proprietary data access, and longitudinal validation studies to assess the real-world effectiveness of TeleRehab DSS and similar systems.

### Conclusions

This scoping review identified 17 smart balance systems (10 investigational and 7 commercial) designed for balance rehabilitation in older adults at risk of falls. The findings highlight key trends in the field, including the dominance of VR-based platforms, widespread motion tracking, and increasing gamification. However, AI integration, real-time feedback, and remote rehabilitation remain underdeveloped areas—features that define TeleRehab DSS. While the SWOT analysis positions TeleRehab DSS as a promising solution with AI-AR personalized balance rehabilitation and integrated clinician monitoring, challenges such as usability remain a critical factor for adoption. Older adults with cognitive impairments or limited digital proficiency may require additional support, including simplified interfaces, adaptive training, and structured clinician-guided engagement. Future research should focus on developing user-friendly interfaces and tailored digital training programs to ensure accessibility across diverse populations.

This review underscores the critical need for ongoing research and development in smart balance telerehabilitation technologies. Future efforts should prioritize large-scale validation studies, user-centered design improvements, and cost-reduction strategies to enhance accessibility, usability, and effectiveness. AI-driven balance rehabilitation systems like TeleRehab DSS can revolutionize fall prevention and rehabilitation for older adults worldwide.

## Supplementary material

10.2196/69589 Multimedia Appendix 1 Database search syntaxes.

10.2196/69589 Multimedia Appendix 2 Characteristics of included studies and commercial systems.

10.2196/69589 Checklist 1 Preferred Reporting Items for Systematic Reviews and Meta-Analyses extension for Scoping Reviews (PRISMA-ScR) checklist.

## Abbreviations

AI: artificial intelligence
AR: augmented reality
HMD: head mounted display
IMU: inertial measurement unit
PRISMA-ScR: Preferred Reporting Items for Systematic Reviews and Meta-Analyses extension for Scoping Reviews
SWOT: strengths, weaknesses, opportunities, and threats
TeleRehab DSS: TeleRehab Decision Support System
VR: virtual reality
