# Supplementary material for: Smart Wearable Technologies for Balance Rehabilitation in Older Adults at Risk of Falls: Scoping Review and Comparative Analysis
Source: JMIR Rehabil Assist Technol. 2025 May 28;12:e69589. doi: 10.2196/69589 (PMC12136182; doi:10.2196/69589)
Supplement: Multimedia Appendix 1 [file rehab-v12-e69589-s001.docx]

***Multimedia Appendix 1****– Database search syntax*

**PUBMED (n=522)**

(Accidental Falls OR dizziness OR dizzyness OR Stroke OR cerebrovascular accident OR brain infarct OR mild cognitive impairment OR cognitive disorders OR cognitive dysfunction* OR vestibular dysfunction OR vestibular disorder OR Long COVID or Post Acute COVID-19 Syndrome OR Long-Haul COVID)

AND (Postural Balance OR balance physiotherapy OR exercise therapy OR vestibular rehabilitation OR balance exercise OR Exercise* OR Physical Therapy OR Physiotherapy OR rehabilitation)

AND (Telerehabilitation OR telemedicine OR technology OR virtual reality OR augmented reality)

AND (Remote Sensing Technology or Wearable Electronic Devices or body sensor* or motion track*)

**COCHRANE (n=138)**

Accidental Falls  OR  dizziness OR dizzyness  OR Stroke OR cerebrovascular accident OR brain infarct OR mild cognitive impairment OR cognitive disorders OR cognitive dysfunction* OR vestibular dysfunction OR vestibular disorder OR Long COVID or Post Acute COVID-19 Syndrome OR Long-Haul COVID
AND
Postural Balance OR balance physiotherapy OR exercise therapy OR vestibular rehabilitation OR balance exercise OR Exercise* OR Physical Therapy OR Physiotherapy OR rehabilitation
AND
Telerehabilitation OR telemedicine OR virtual reality OR augmented reality 
AND
Remote Sensing Technology or Wearable Electronic Devices or body sensor* or motion track*

**MEDLINE (n=3)**
dizziness/ or Accidental Falls/ or dizzyness/ or Stroke/ or cerebrovascular accident/ or brain infarct/ or mild cognitive impairment/ or cognitive disorders/ or cognitive dysfunction*/ or vestibular dysfunction/ or vestibular disorder/ or Long COVID/ or Post Acute COVID-19 Syndrome/ or Long-Haul COVID/
AND
Postural Balance/ or balance physiotherapy/ or Exercise Therapy/ or vestibular rehab*/ or Exercise/ or physical therapy/ or Physiotherapy/ or rehabilitation/
AND
Telerehabilitation/ or telemedicine/ or Augmented Reality/ or Virtual Reality/
AND
Remote Sensing Technology/ or Wearable Electronic Devices/ or body sensor* or motion track*
