# Supplementary material for: Smart Wearable Technologies for Balance Rehabilitation in Older Adults at Risk of Falls: Scoping Review and Comparative Analysis
Source: JMIR Rehabil Assist Technol. 2025 May 28;12:e69589. doi: 10.2196/69589 (PMC12136182; doi:10.2196/69589)
Supplement: Multimedia Appendix 2 [file rehab-v12-e69589-s002.docx]

***Multimedia Appendix 2–*** *Characteristics of included studies and commercial systems*

**Table 1**. Characteristics of included studies

| **Author (year) *Country*** | **Title** | **Study Aim** | **Population of use** | **Intervention/system summary** |
| --- | --- | --- | --- | --- |
| Rosiak et al. (2018) [25]  *Poland* | Evaluation of the effectiveness of a Virtual Reality-based exercise program for Unilateral Peripheral Vestibular Deficit | To assess the effectiveness of a low-cost hybrid VR based vestibular rehabilitation program | Adults (18+) with peripheral vestibular dysfunction | Hybrid VR unit including a force plate, upper body IMU, and a flat screen display. |
| Liao et al. (2015) [26]  *Taiwan* | Virtual Reality-Based Wii Fit Training in Improving Muscle Strength, Sensory Integration Ability, and Walking Abilities in Patients with Parkinson's Disease: A Randomized Control Trial | Examine the effects of VR-based training in improving muscle strength, sensory integration ability, and walking abilities in patients with Parkinson’s Disease through a [randomized controlled trial](https://www.sciencedirect.com/topics/medicine-and-dentistry/randomized-controlled-trial). | Adults with Parkinson’s disease | A motion controlled virtual avatar-based game using the Nintendo Wii Fit Plus gaming system and computer interface. |
| Ku J et al. (2019) [27]  *Korea* | Three-Dimensional Augmented Reality System for Balance and Mobility Rehabilitation in the Elderly: A Randomized Controlled Trial | Evaluate the clinical efficiency of a novel three-dimensional interactive augmented reality system for balance and mobility rehabilitation. | Older adults (n=36) (55-80) who could ambulate independently & stand on one leg | A 3d AR system for balance and mobility rehabilitation with full-body motion tracking & interaction with virtual objects on a computer screen display. |
| Guo et al. (2023) [16]  *China* | Clinical Study of a Wearable Remote Rehabilitation Training System for Patients with Stroke: Randomized Controlled Pilot Trial | Evaluate the effectiveness and safety of the remote rehabilitation training system for nonphysician-supervised motor rehabilitation training of patients with stroke through a clinical trial study | Stroke in patients with limb motor dysfunction | A remote intelligent rehabilitation training system based on wearable devices (2 IMU + glove IMU) and human-computer interaction training tasks |
| Yun et al. (2020) [29]  *Korea* | Cognitive Training Using Fully Immersive, Enriched Environment Virtual Reality for Patients with Mild Cognitive Impairment and Mild Dementia: Feasibility and Usability Study | Investigate the feasibility and usability of cognitive training using fully immersive VR programs in enriched environments with physiatrists, occupational therapists (OTs), and patients with mild cognitive impairment and mild dementia | 10 physiatrists, 6 OTs, and 11 older adults (65+) with mild cognitive impairment | Cognitive training system using virtual reality through a VR HMD (HTC Vive), wrist IMU, and AI hand position estimation using a wrist-worn camera. |
| Proffitt et al. (2018) [21]  *USA* | Safety and Feasibility of a First-Person View, Full-Body Interaction Game for Telerehabilitation Post-Stroke | To test initial safety among fourteen participants without disabilities (age 30 ± 8.8 years) engaged in a game-based task using the Microsoft Kinect® with a first-person view using the Oculus Rift. | Post-stroke adults (18+) | A rehabilitation virtual reality gaming system for post-stroke patients as a home exercise program. Using Microsoft Kinect to track full body movements during tasks via a VR HMD (Oculus Rift). |
| Khushnood (2019) [22]  *Pakistan* | Virtual reality-based training for subjects with stroke | Investigate the X-box Kinect under physiotherapists supervision on Balance, fall risk and functional independence in stroke patients. | Stroke >3-months post older adults aged 45-65. | X-Box Kinect VR -based games with a body motion sensor. |
| Silva Bessa (2019) [23]  *Brazil* | Influence of a Virtual Reality treatment on Postural Balance of patients with Stroke | Evaluations will collect data on clinical characterization, cognition, spasticity, cerebral activation pattern, neurological impairment, quality of life, functionality, balance and gait. Virtual Reality | First stroke, aged 20-75, max 2 on modified Ashworth scale, | Lower limb strengthening plus a kinesiotherapy protocol with a balance of motion and sensory‐motor demand similar to VR exercises.  Experimental Group will perform the same strengthening plus a VR protocol based on body balance. |
| Silva Soares (2019) [24]  *Brazil* | The use of videogame as a resource for the recovery of post-stroke patients | To evaluate motor control regarding body balance in an objective (baropodometry) and qualitative way (Time Testing up and go), use of the paretic limb (Fugl-Meyer Scale and Motor Assessment Scale), and motivation of a group of participants using virtual reality. | Post-stroke patients age 35+, hemiparesis, ability to ambulate, no physio. | Physiotherapeutic treatment using VR using adventure games that require limbs and trunk movements, stimulating static and dynamic balance and motor coordination to accomplish the proposed task. |
| Zeigelboim et al. (2021) [28]  *Brazil* | Balance rehabilitation with a virtual reality protocol for patients with hereditary spastic paraplegia: protocol for a clinical trial | To determine the benefits of vestibular rehabilitation involving virtual reality by comparing pre intervention and post intervention assessments in individuals with hereditary spastic paraplegia. | Adults (18+) with hereditary spastic paraplegia (HSP) | Virtual reality vestibular rehabilitation using the Nintendo Wii console (Wii Remote and Wii Balance Board) on a TV display. |

HMD: head mounted display; VR: virtual reality; AR: augmented reality;

**Table 2.** Commercially available systems in the market that met inclusion criteria.

| **Product/Company Name** | System summary | Population of use |
| --- | --- | --- |
| **TeleRehab DSS** | The TeleRehab DSS platform, evolved from its predecessor, HOLOBalance[31], provides am individualised balance rehabilitation programme. The TeleRehab DSS comprises a depth camera, lightweight AR headset that displays the hologram, body-worn sensors to record movements and a heart rate sensor (see Figure 1). It also provides real-time feedback on individual’s performance of AR delivered multisensory exercises, cognitive training and exergames. | Adults 40-80 years of age, at risk of falls due to either: stroke, MCI, vestibular dysfunction or long covid-19 |
| **HoloBalance** | The HOLOBalance system provides an evidence-based balance training program delivered to patients in their home environment using a novel technological approach including an augmented reality virtual physiotherapist, exergames and a remote monitoring system. | Older adults (50-80 years of age) at risk of falls |
| **Home balance** | Smart balance system using a balance board, tablet display, and wearable motion tracker to provide distance balance therapy under clinician supervision. | Patients with balance disorders caused by neurological conditions, injuries, or aging-related issues. |
| **Reflexion Health - VERA** | Home based avatar-led rehab system with integrated sensors and display for minimal set up. Video capture allows real-time feedback from guiding clinicians. | Patients with physical therapy |
| **XRhealth** | Virtual clinic providing occupational, physical & psychological therapies through VR programs. Patient receives a VR headset & handheld sensor, a personalised care plan and video conferencing to a clinician. | Variety of health conditions, including mental health, physical rehabilitation, and cognitive improvement, providing both patients and providers with advanced options for treatment and therapy. |
| **Evolv Rehab** | Evolv Rehab kit’ fully integrated sensor & console system for remote rehabilitation. The system plugs into a display & operates with a single start button and internet connection, requiring little technology experience for the use. | Older adults (65+) 'various neuromotor impairments' |
| **Jintronix** | Virtual training involving balance exercise accessible to patient at any time. | Older adults (65+) |
| **TRAK** | An AI-powered telerehabilitation application for the assessment & prescription of digital physiotherapy. | Variety of health conditions |
